# HDAC6 promotes inflammation in lupus nephritis mice by regulating transcription factors MAFF and KLF5 in renal fibrosis

**DOI:** 10.1080/0886022X.2024.2415517

**Published:** 2024-10-16

**Authors:** Meihui Deng, Xiao Tan, Xiaojie Peng, Weimin Zheng, Rui Fu, Shanshan Tao

**Affiliations:** a Department of Nephrology, Jiangxi Provincial Children’s Hospital, Nanchang, Jiangxi, P.R. China; b Department of Hematology, Jiangxi Provincial Children’s Hospital, Nanchang, Jiangxi, P.R. China

**Keywords:** Lupus nephritis, renal fibrosis, HDAC6, MAFF, KLF5, MRL/lpr mice

## Abstract

**Aim:**

This study explored the effect and mechanism of MAFF and HDAC6 on renal fibrosis and inflammation in lupus nephritis (LN).

**Methods:**

IL-33 treated renal epithelial cells and MRL/lpr mice were respectively used for *in vitro* and *in vivo* experiments. The expressions of HDAC6, MAFF, and KLF5 were measured in cells and renal tissues. Before and after cell transfection, the morphological changes in renal tissues were observed using Hematoxylin and eosin (H&E) and Masson staining. The proteinuria, serum creatinine (SCr), blood urea nitrogen (BUN), and double-stranded DNA (dsDNA) levels were detected by biochemical analysis. The expressions of fibrosis and inflammation related proteins (including α-SMA, Vimentin, IL-1β, IL-6, and TNF-α), p65, and iNOS were also detected. The relationship among MAFF, HDAC6, and KLF5 was determined by chromatin immunoprecipitation and dual luciferase reporter gene assay.

**Results:**

Renal tissues and cell models had elevated expressions of HDAC6 and KLF5, and decreased MAFF expression. HDAC6 suppression or MAFF overexpression led to suppression of proteinuria, SCr, BUN, and dsDNA levels, as well as attenuation of inflammatory infiltration and collagen deposition. HDAC6 can suppress MAFF expression *via* deacetylation to abolish its suppression of KLF5 expression, thus increasing KLF5 expression. *In vivo* and *in vitro* experiments showed the suppressive effect of HDAC6 suppression on renal fibrosis and inflammation can be abolished by KLF5 overexpression.

**Conclusion:**

HDAC6 suppresses MAFF expression *via* deacetylation to elevate KLF5 expression, which consequently enhances fibrosis and inflammatory response in LN.

## 1. Introduction

Lupus nephritis (LN) is a kind of glomerulonephritis and occurs in nearly 50% of the autoimmune disease systemic lupus erythematosus (SLE) [1,2]. As a major contributor to renal injury in SLE, LN is associated with considerable morbidity and mortality in SLE cases [3]. The immune complex depositions in the kidney are the main character of LN, which results in inflammatory and immune response that eventually leads to renal fibrosis [4,5]. Currently, increasing approaches have been proposed for the treatment of LN, including immunosuppressive therapy, typically with mycophenolate mofetil or cyclophosphamide in class III-IV LN, and Food and Drug Administration (FDA)-approved belimumab and voclosporin [6,7]. Unfortunately, more than 20% of LN patients progressed to end-stage renal disease (ESRD) despite treatment [8], but there are currently limited data concerning the long-term prognosis of pediatric LN [9]. The incidence of LN in children is much higher than that in adults and the disease progression is much worse in childhood instead in adulthood [10]. Although early response to treatment is coupled with favorable renal outcomes in adults [11], early predictors of renal function impairment are lacking for pediatric patients.

Histone deacetylases (HDACs) are mainly response for the regulation of gene expression through remodeling chromatin structure, whose deregulated expression has been well reported in multiple diseases, including solid tumor and inflammatory disorders [12,13]. As the largest member of HDAC family, HDAC6 has been previously researched for its role in regulating cellular pathways [14]. Moreover, HDAC6 inhibitor Tubastatin A was found to suppress fibrosis and inflammation in hypertensive mice [15] and attenuate renal tubular damage in acute kidney injury [16], this suggests that HDAC inhibition has beneficial effects in renal fibrosis (Supplementary Figure 1). HDAC6 inhibitors were used to treat cancers without apparent toxicity, such as breast cancer [17], gastrointestinal cancer [18], and non-small cell lung cancer [19]. In mouse renal fibrosis models, suppression of HDAC6 expression led to suppressed fibroblast activation and extracellular matrix (ECM) deposition by blocking α-smooth muscle actin (α-SMA), collagen type III, and fibronectin expressions [20]. Nevertheless, the mechanism by which HDAC6 mediates renal fibrosis in LN remains much to be determined.

MAFF is a transcription factor whose expression was found in both human kidney graft biopsy and rodent ­ischemia/reperfusion injury (IRI) induced renal injury [21]. In addition to that, suppression of MAFF may expand podocyte injury, consequently leading to the destruction of renal structure and event renal failure [22]. Analysis of GSE51921 and GSE112493 datasets further identified the deregulated expression of MAFF in LN. However, less information was available regarding the possible effect of MAFF in LN. H3K27ac peaks were detected in the promoter of MAFF using UCSC database, suggesting the transcription of MAFF may be regulated by histone acetylation. Moreover, JASPAR found the binding sites between MAFF and Kruppel-like factor 5 (KLF5). Considering the finding of our previous study that KLF5 promotes renal fibrosis in MRL/lpr mice [23], it is reasonable to speculate the possible effect of HDAC6/MAFF/KLF5 axis in LN. In this study, we explored the role of the HDAC6/MAFF/KLF5 axis in renal fibrosis and inflammation using both *in vivo* and *in vitro* experiments with the expectation to provide a theoretical basis for proposing new treatment approaches for LN patients, especially for pediatric patients.

## 2. Materials and methods

The methods of this study are shown in Supplementary Figure 2.

### 2.1. Bioinformatics analysis

GSE32591 or GSE112493 datasets from GEO (http://www.ncbi.nlm.nih.gov/geo) were downloaded for limma analysis using SangerBox. Notably, GSE32591 dataset analysis did not distinguish between tubulointerstitial and glomerulus, only between LN patient kidney tissue samples and healthy control tissue samples. The statistical significance was set at |logFC| > 1 and *p* value <0.05. The differentially expressed genes (DEGs) were presented using volcano map.

### 2.2. Mouse feeding

The mouse experiment was conducted after the experiment design was approved by the ethical committee of our hospital and based on the regulations of laboratory animal management. Male MRL/lpr (*n* = 36) and BALB/c (*n* = 6) mice all aged 7 weeks were purchased from Junke Biological Co., Ltd. (Nanjing, China). Mice were housed in specific pathogen free (SPF) cages with the temperature of 21–25 °C, humidity of 50–65%, and light/dark cycle of 12:12 h. Food and water were accessible to all mice.

### 2.3. Groupings and corresponding treatment

After adapting feeding for one week, MRL/lpr mice were randomly grouped into MRL/lpr (*n* = 6), oe-MAFF (oe-MAFF injection *via* tail vein, *n* = 6), oe-NC (oe-NC injection *via* tail vein, *n* = 6), sh-NC + oe-NC (oe-NC + sh-NC injection *via* tail vein, *n* = 6), sh-HDAC6 + oe-NC (sh-HDAC6 + oe-NC oe-NC + sh-NC injection *via* tail vein, *n* = 6) and sh-HDAC6 + oe-KLF5 groups (sh-HDAC6 + oe-KLF5 injection *via* tail vein, *n* = 6), whereas BALB/c mice were used as controls. The adenoviral vector was constructed based on following steps: interference or overexpression sequence of the target gene was designed and synthesized, and the amplified sequence was inserted into the linearized adenoviral expression vector (pAV-EGFP-U6). The recombinant plasmid was checked for positive clone by sequencing. Then, 293 T cells were transfected with the vector plasmid and the viral packaged plasmid carrying the target gene for cell culture for 5 days, during which the medium was changed to complete medium after 6 h of transfection. On day 6, cell supernatant was collected, and the purified and concentrated solution was subjected to viral titer detection. The titer of adenoviral vector is (1 × 10^9^ pfu/100 μl) [24]. The transfection was continued for 12 weeks, twice a week. On week 13, mice were euthanized by cervical dislocation. Blood from the right atrium of mice was collected to extract the supernatant for further analysis. The renal tissues were dissected on ice with part of the tissues for morphology detection and the rest for storage at −80 °C.

### 2.4. Biochemical analysis

The urine of mice 24 h before euthanasia was collected by tail lifting method and the proteinuria was measured using a microreader (Bio-Rad, Hercules, CA, USA) at 595 nm after Coomassie brilliant blue G250 (Solarbio, Beijing, China) staining.

Serum of 20-week-aged mice was collected to determine serum creatinine (SCr) and blood urea nitrogen (BUN) levels. The double-stranded DNA (dsDNA) level was determined using mouse anti dsDNA antibody ELISA kit (Thermo Fisher Scientific, Waltham, MA, USA).

### 2.5. Hematoxylin and eosin (H&E) staining

Renal tissues were fixed in 4% formaldehyde solution, followed by hydration, waxing, and embedding before made into slices (4 μm). The slices were stained with H&E and hydrated for transparency. The sealed slices were observed under a microscope (Olympus, Tokyo, Japan). As previously described, the severity of LN was evaluated on a scale of 0–6 [25], and glomerular lesions were assessed on a scale of 0–4 [26].

### 2.6. Masson staining

Dewaxed slices were stained with Hematoxylin for 30 s and washed with distilled water, followed by washing with blue promoting solution for 3 min, rinsing with Lichun Red Acid Magenta for 3 min, and treating with phosphomolybdate for 5 min. Then the slices were stained with aniline blue for 5 min and washed in running water before differentiation, with eosin staining and dehydration in gradient alcohol. After permeabilization and sealing, slices were observed under a light microscope (Olympus).

### 2.7. Immunohistochemistry (IHC)

Slices were baked 30 min for dewaxing using xylene and then reacted with 3% H_2_O_2_ for 10 min at room temperature. Normal goat serum was added to block unspecific response, after which the slices were incubated with primary normal goat serum (ab5831, 1:100, Abcam, Cambridge, MA, USA) at 4 °C for overnight. The secondary antibody was incubated at room temperature for 1 h before 3,3′-diaminobutyric acid (DAB) solution was added for color development for 1–3 min. Cell nucleus was stained using eosin for 3 min and slices were dehydrated, permeabilized, and sealed. A microscope (×200, Olympus) was used for morphology observation with 10 randomly selected fields (at least 100 cells). IHC score was assessed using Image J software based on the percentage of positive cells and staining intensity of cells [27].

### 2.8. Cell culture

Mouse renal epithelial cells (Procell Life Science & Technology Co., Ltd., Wuhan, China) were cultured in Roswell Park Memorial Institute (RPMI)-1640 culture medium containing 10% fetal bovine serum (FBS) and 100 mg/mL penicillin-streptomycin (Gibco, Grand Island, NY, USA) at 37 °C with 5% CO_2_. The culture medium was refreshed every 2 days.

LN fibrosis or inflammatory cell models were established using IL-33 (100 ng/ml, Sigma, St. Louis, MO, USA) based on the methods described in a previous study [28].

### 2.9. Cell transfection

sh-HDAC6 (sequence: CCTTGCTGGTGGCCGTATTAT), sh-MAFF (sequence: GACTCTTCCACACTCTTATTT), oe-MAFF, oe-KLF5 and their corresponding negative controls were all purchased from VectorBuilder (Shanghai, China). The concentration of vectors was 100 nM [29] and transfection was performed using Lipofectamine 2000 reagent (Invitrogen, Carlsbad, CA, USA) in accordance with manufacturer’s instruction.

### 2.10. Reverse transcription quantitative polymerase chain reaction (RT-qPCR)

Total RNA from cells and tissues were extracted using Trizol method, whose purity was detected using NanoDrop spectrophotometer (Thermo Fisher Scientific). RT was performed using kit (Thermo Fisher Scientific) and the detection was performed using Biosystems 7300 real time PCR system (ABI, Foster City, CA, USA) and SYBR GreenMix (Takara, Japan). Each reaction was set 3 duplicates and data were analyzed using 2^−ΔΔCt^ method. The expression of the target gene was relative to that of glyceraldehyde-3-phosphate dehydrogenase (GAPDH). The primers and sequences are listed in Table 1.

**Table 1.** Primer sequences.

Name of primer	Sequences (5′-3′)
HDAC6-F	TGCAGGAGGTGGAGTTGAGT
HDAC6-R	GAAGAATCTTGGCCGGTGGA
MAFF-F	GTTCTCCTAGGCTGAGGATGTG
MAFF-R	ATCAGCGCTTCATCCGACA
KLF5-F	CACCGGATCTAGACATGCCC
KLF5-R	ACGTCTGTGGAACAGCAGAG
GAPDH-F	CCCTTAAGAGGGATGCTGCC
GAPDH-R	ACTGTGCCGTTGAATTTGCC
p65-F	CCTCGGGACAAACAGCCTC
p65-R	TGCTTCGGCTGTTCGATGAT
iNOS-F	CAACAGGGAGAAAGCGCAAA
iNOS-R	GGCCTTGTGGTGAAGAGTGT

F: forward; R: reverse.

### 2.11. Western blot

Proteins in the renal tissues and cells were treated with Radio-Immunoprecipitation assay (RIPA) lysis, followed by bicinchoninic acid (BCA) kit detection on protein concentration. Then polyacrylamide gel electrophoresis was performed to isolate the proteins which were transferred into polyvinylidene fluoride (PVDF) membranes. The proteins were then blocked with 5% skimmed milk powder to eliminate the influence of unspecific response. The following antibodies were used as primary antibody for incubation at 4 °C overnight: α-SMA (1:1000, ab5831, Abcam), Vimentin (1:1000, ab92547, Abcam), HDAC6 (1:1000, PA1-41056, Thermo Fisher Scientific), MAFF (1:1000, ab227721, Abcam), KLF5 (1:2000, ab137676, Abcam), H3K27ac (1:2000, ab4729, Abcam), interleukin-1beta (IL-1β) (1:2000, ab254360, Abcam), IL-6 (1:1000, ab259341, Abcam), tumor necrosis factor-α (TNF-α) (1:1000, ab183218, Abcam), and GAPDH (1:2000, ab8245, Abcam). The membranes were washed in Tris-buffered saline-tween (TBST) for 3 × 10 min before horseradish peroxidase (HRP) labeled secondary antibody immunoglobulin G (IgG) was added for further incubation. Then enhanced chemiluminescence (ECL) color development solution (P0018FS, Beyotime, Shanghai) was added and the membranes were detected under a chemiluminescence imaging system (Bio-Rad). Data were analyzed using Quantity One v4.6.2 software.

### 2.12. Immunofluorescence

Digested cells were evenly placed in the plate with 2 × 10^5^ cells per well. Once cells reached the confluency of 60–80%, cells were washed on phosphate-buffered saline (PBS) for 3 times, each for 5 min, and then fixed in 4% paraformaldehyde for 15 min, followed by PBS washing for 3 times, each for 5 min. The 5% serum was used for cell blocking for 1 h. Cells were washed in PBS for 3 times, each for 5 min before and after permeabilization with PBS containing 0.2% Triton-×100. Primary antibodies of α-SMA (1:100, ab5831, Abcam) and Vimenntin (1:200, ab92547, Abcam) were added for incubation for overnight at 4 °C. The slices were washed in PBS before fluorescence marked secondary antibody was added for incubation in the dark for 1 h. Afterward, 4′,6-diamidino-2-phenylindole (DAPI) was used for nucleus staining for 15 min after slices were washed in PBS. The images were observed and photographed under a florescent microscope (Olympus).

### 2.13. Chromatin immunoprecipitation (ChIP) assay

Cells were treated with paraformaldehyde for 10 min and sonicated into chromatin fragment, followed by 4 °C centrifugation at 12,000 g for 10 min with the supernatant being collected in two tubes. The tubes were respectively reacted with negative control antibody IgG (ab172730, Abcam) or specific antibodies for anti-HDAC6 (PA1-41056, Thermo Fisher Scientific), anti-H3K27ac (ab4729, Abcam), MAFF (ab227721, Abcam), and IgG (ab182931, Abcam) at 4 °C for overnight. The DNA-protein complex was reacted with Protein Agarose/Sepharose at 12,000 g for 5 min. The supernatant was removed and the unspecific complex was abandoned through washing. The complex was de-crosslinked at 65 °C and the DNA fragment was extracted using phenol-chloroform extraction method. The purified DNA fragment was detected using PCR. The primer sequences for MAFF promoter are: F: 5′-AGACCCAGGGAGTCTGG GA-3′, R: 5′-CCACAGAGCCATCTCACCTG-3′; the sequences for KLF5 promoter are: 5′-ACGGTTAGACCATTCTAGGAAAT-3′, R: 5′-GCATACACTATCGGAAACAA CC-3′.

### 2.14. Dual luciferase reporter gene assay

The binding sites of MAFF and KLF5 were predicted in JASPER (https://jaspar.genereg.net/). The wt-KLF5 and mut-KLF5 reporter gene plasmid were constructed accordingly and transfected into renal epithelial cells with oe-NC or oe-MAFF for incubation at 37 °C for 48 h with 5% CO_2_. The collected cells were washed with PBS and treated with lysis buffer before centrifugation to collect the supernatant. The fluorescent intensity was measured with dual luciferase reporter gene kit (Promega, Madison, WI, USA).

### 2.15. Statistical analysis

GraphPad 8.0 was used for data analysis with all data expressed as mean ± standard deviation. *t*-Test was used for comparison between two groups, while one-way analysis of variance and Tukey’s multiple comparisons test were used for comparison among multiple groups. The statistical significance was set at *p*-value <0.05.

## 3. Results

### 3.1. MAFF overexpression attenuates renal fibrosis and inflammation in LN mouse

**Figure 1.**
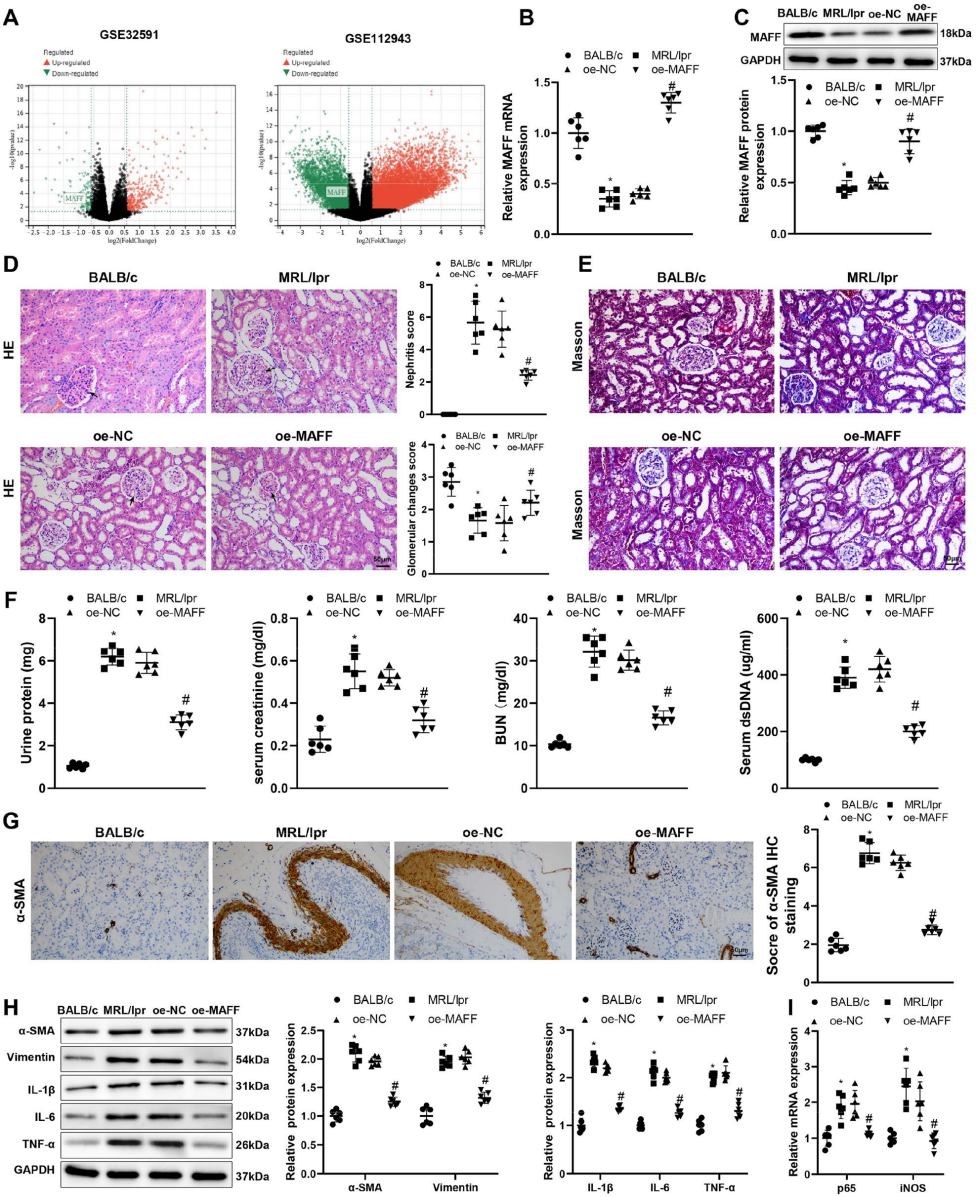
MAFF overexpression attenuates renal fibrosis and inflammatory response in LN mouse, (A) MAFF expression data from GSE32591 and GSE112493 datasets (for GSE32591 dataset, LN patients = 64, healthy controls = 30; for GSE112493 dataset, LN patients = 14, healthy controls = 7); (B,C) MAFF expression in MRL/lpr mice or BALB/c mice was detected by RT-qPCR and Western blot; (D,E) morphology observation of renal tissues using H&E and Masson staining; (F) biochemical analysis on proteinuria, SCr, BUN and dsDNA levels; (G) IHC detecting α-SMA expression; (H) protein expressions of α-SMA, Vimentin, IL-1β, IL-6, and TNF-α were detected using Western blot. (I) The mRNA levels of p65 and iNOS were tested using RT-qPCR. *N* = 6 for mice experiments (B–I). Data were expressed as mean ± standard deviation. One-way analysis of variance and Tukey’s multiple comparisons test were used for comparison among multiple groups. *p < 0.05, when compared with BALB/c group; ^#^*p* < 0.05, when compared with oe-NC group. LN: lupus nephritis; BUN: blood urea nitrogen; dsDNA: double-stranded DNA; SCr: serum creatinine; IHC: immunohistochemistry; H&E: hematoxylin and eosin staining.

The analysis of MAFF expression in GSE32591 and GSE112493 datasets found decreased MAFF expression in LN (Figure 1(A)). Then the expressions of MAFF in the renal tissues of MRL/lpr mice were detected by both RT-qPCR and western blot. The results showed MAFF expression was deceased in MRL/lpr mice, compared with BALB/c mice (Figures 1(B,C), *p < 0.05). The morphology observation on renal tissues showed mice in MRL/lpr group had proliferated glomerular mesangial cells, infiltrated inflammatory cells, and increased collagen deposition, instead of BALB/c mice (Figures 1(D,E), *p < 0.05). The biochemical analysis showed proteinuria, SCr, BUN, and dsDNA levels in MRL/lpr mice were all increased (Figure 1(F), *p < 0.05). IHC detecting the expression level of α-SMA demonstrated elevated α-SMA expression in renal tissues of MRL/lpr mouse rather than BALB/c mouse (Figure 1(G), *p < 0.05).

The proteins related to fibrosis (α-SMA and Vimentin) and inflammation (IL-1β, IL-6, and TNF-α) were assessed by western blot. The results found enhanced fibrosis and inflammatory response in renal tissues of MRL/lpr mice (Figure 1(H), *p < 0.05). As key genes for oxidative stress [30,31], p65 and iNOS were detected to be enhanced in MRL/lpr mouse tissues when compared to BALB/c mouse tissues, elicited by RT-qPCR (Figure 1(I), *p < 0.05). The transfection of oe-MAFF in MRL/lpr mice resulted in the elevation MAFF expressed and alleviation of renal morphological changes in addition to attenuation of renal fibrosis and inflammation (Figures 1(B–I), ^#^*p* < 0.05). Above results demonstrated MAFF was decreased in renal tissues of LN mice whereas overexpression of MAFF can attenuate renal fibrosis and inflammation of LN mice.

### 3.2. MAFF overexpression suppresses fibrosis and inflammatory response in renal epithelial cells

**Figure 2.**
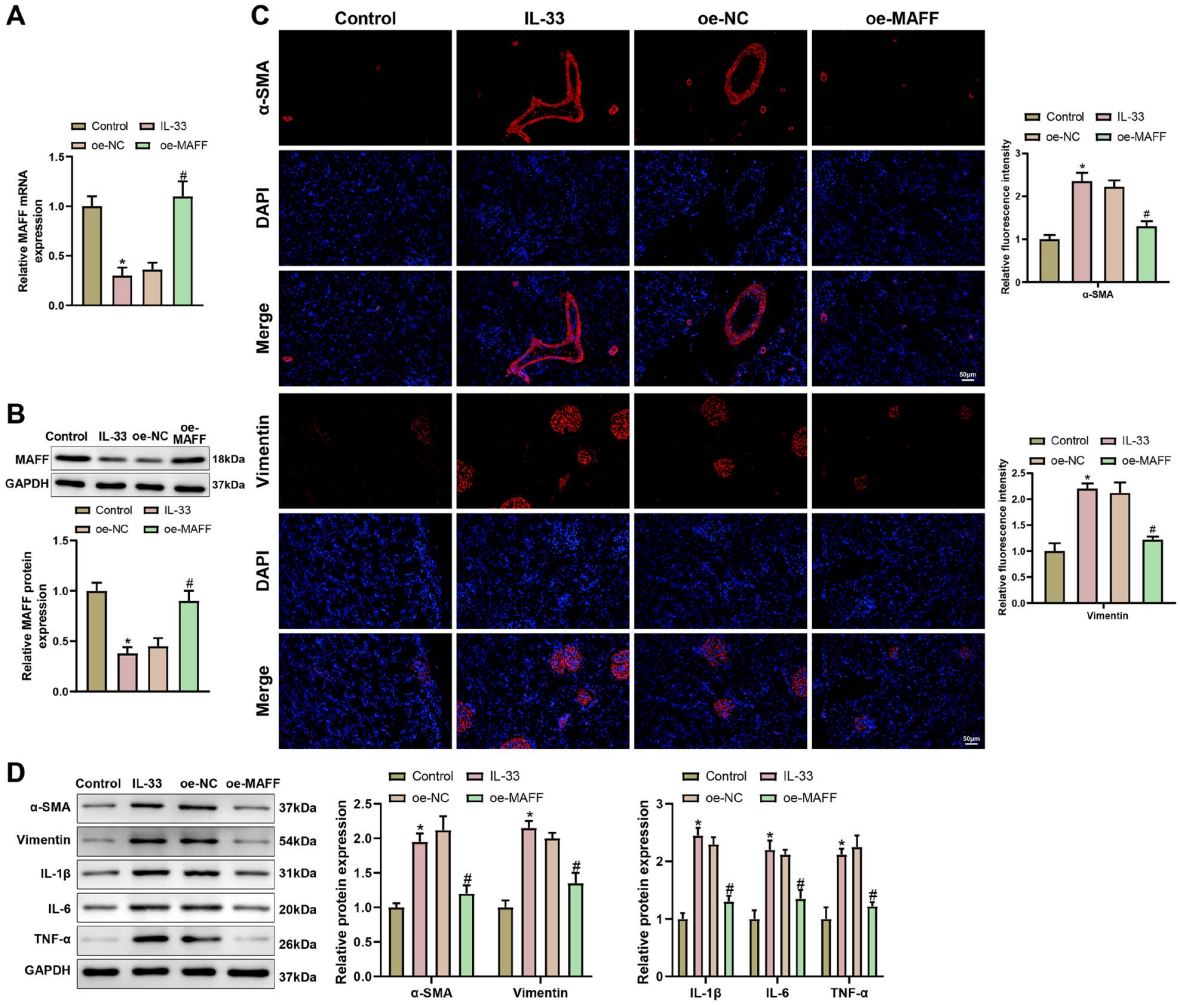
MAFF overexpression attenuates fibrosis and inflammation in IL-33 induced renal epithelial cells, (A,B) MAFF mRNA and protein expressions were detected after oe-MAFF was transfected into renal epithelial cells; (C) the expression levels of α-SMA and vimentin were measured by immunofluorescence; (D) the expressions of fibrosis and inflammation related proteins, including α-SMA, Vimentin, IL-1β, IL-6, and TNF-α were detected by Western blot. Data were expressed as mean ± standard deviation. *N* = 3 for cellular experiments. One-way analysis of variance and Tukey’s multiple comparisons test were used for comparison among multiple groups. *p < 0.05, when compared with control group; ^#^*p* < 0.05, when compared with oe-NC group.

IL-33 (100 ng/ml) was used to induce fibrosis and inflammation in renal epithelial cells [28]. To determine the effect of MAFF on renal fibrosis and inflammation, we first detected MAFF expression after IL-33 treatment. RT-qPCR, western blot, and immunofluorescence demonstrated IL-33 treatment inhibited MAFF expression (Figures 2(A,B), *p < 0.05), but increase α-SMA, Vimentin, IL-1β, IL-6, and TNF-α expression (Figures 2(C,D), *p < 0.05), compared with control group. However, the effect of IL-33 treatment could be reversed by MAFF overexpression (Figures 2(A–D), *p < 0.05). In summary, overexpressed MAFF alleviated fibrosis and inflammatory response in renal epithelial cells.

### 3.3. HDAC6 regulates MAFF transcription to mediate fibrosis and inflammation in renal epithelial cells

Although MAFF overexpression can suppress renal fibrosis and inflammation, the mechanism herein remains to be determined. UCSC database identified H3K27ac peaks in MAFF promoter (Supplementary Figure 3(A)), indicating the transcription of MAFF is regulated by histone acetylation. HDACs play paramount roles in regulating gene expression [32] and suppression of HDAC6 expression was proved to attenuate LN [33,34]. Therefore, we performed a series of functional assays (RT-qPCR, western blot, and ChIP) and found that HDAC6 may decrease MAFF expression through deacetylation (Supplementary Figures 3(B–I)).

**Figure 3.**
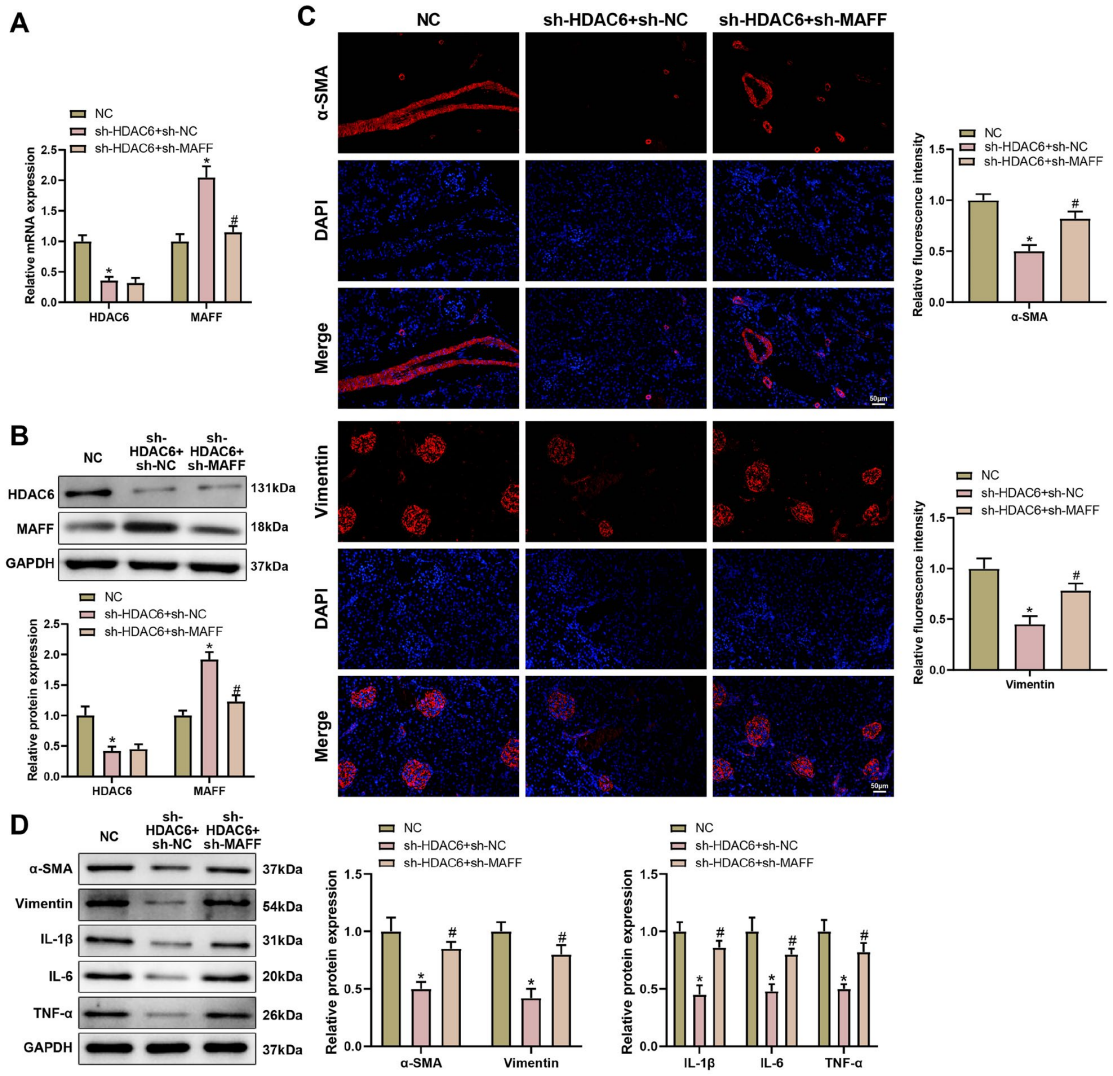
HDAC6 Decreased MAFF expression *via* deacetylation to mediate fibrosis and inflammatory response in renal epithelial cells, the sh-HDAC6 or/and sh-MAFF were transfected into IL-33 treated renal epithelial cells. (A,B) RT-qPCR and Western blot detected the expressions of MAFF and HDAC6; (C) immunofluorescence detected the expressions of α-SMA and vimentin; (D) western blot detected the proteins related to fibrosis and inflammation, including α-SMA, Vimentin, IL-1β, IL-6, and TNF-α. Data were expressed as mean ± standard deviation. *N* = 3 for cellular experiments. One-way analysis of variance and Tukey’s multiple comparisons test were used for comparison among multiple groups. *p < 0.05, when compared with NC group; ^#^*p* < 0.05, when compared with sh-HDAC6 + sh-NC group. NC group is the negative control for sh-HDAC6 group and sh-MAFF group.

After sh-HDAC6 or/and sh-MAFF were transfected into IL-33 treated renal epithelial cells, the expression changes of MAFF were detected. RT-qPCR and western blot showed in response to sh-HDAC6 and sh-MAFF transfection, the MAFF expression was decreased while no change in HDAC6 expression was detected (Figures 3(A,B), ^#^*p* < 0.05). The measurement of fibrosis and inflammation showed attenuated fibrosis and inflammation in response to sh-HDAC6 transfection (Figures 3(C,D), ^#^*p* < 0.05), and enhanced fibrosis and inflammation condition in cells co-transfected with h-HDAC6 and sh-MAFF (Figures 3(C,D), ^#^*p* < 0.05). Collective results demonstrated that HDAC6 reduced MAFF transcriptional expression through deacetylation, thus affecting renal epithelial cell fibrosis and inflammation.

### 3.4. HDAC6 mediates fibrosis and inflammation in renal epithelial cells through MAFF/KLF5 axis

MAFF working as a transcription factor can activate or suppress its downstream genes [35,36]. KLF5 was found to be increasingly expressed in LN [37]. JASPER database found the binding sites of MAFF with KLF5 promoter (Supplementary Figure 4(A)). Therefore, KLF5 was speculated as a downstream factor for HDAC6/MAFF axis in LN. Accordingly, results of RT-qPCR, western blot, ChIP, and dual luciferase reporter gene assay revealed that the transcription factor MAFF bound to the KLF5 promoter and inhibited KLF5 transcription expression (Supplementary Figures 4(B–G)).

**Figure 4.**
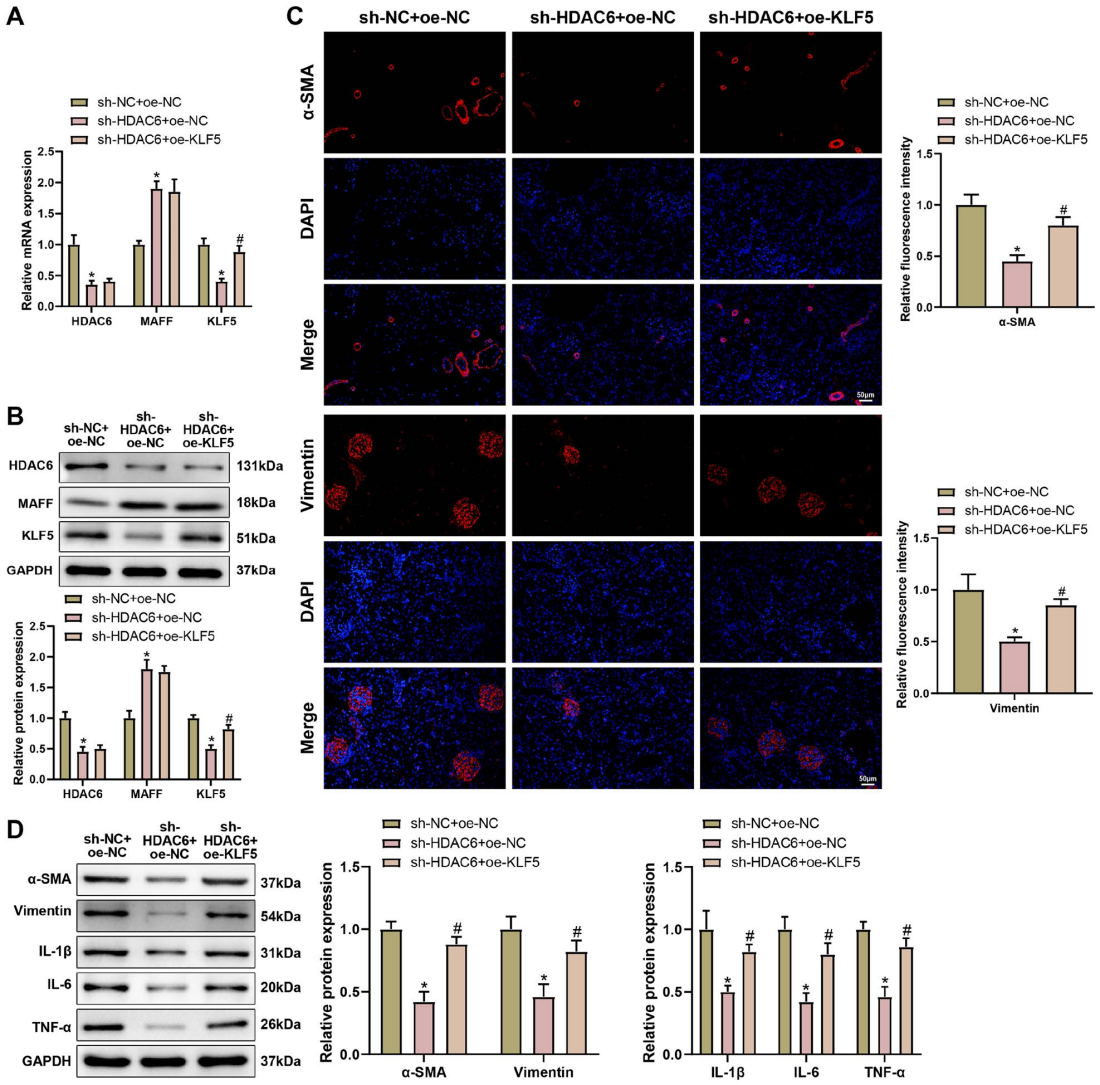
KLF5 Overexpression reversed the effect of HDAC6 suppression on fibrosis and inflammation in renal epithelial cells, (A,B) expressions of HDAC6, MAFF, and KLF5 were detected by RT-qPCR and Western blot; (C) α-SMA and vimentin expressions was detected by immunofluorescence; (D) Western blot was applied to detect the expressions of fibrosis and inflammation related proteins, α-SMA, Vimentin, IL-1β, IL-6, and TNF-α. Data were expressed as mean ± standard deviation. *N* = 3 for cellular experiments. One-way analysis of variance and Tukey’s multiple comparisons test were used for comparison among multiple groups. *p < 0.05, when compared with sh-NC + oe-NC group; ^#^*p* < 0.05, when compared with sh-HDAC6 + oe-NC group.

To clarify the implication of KLF5 on the HDAC6/MAFF axis in LN, we co-transfected sh-HDAC6 with oe-KLF5 into IL-33 induced renal epithelial cells to determine the alternation on expressions of HDAC6, MAFF, and KLF5. The RT-qPCR and western blot found compared with sh-NC + oe-NC group, sh-HDAC6 + oe-NC group had decreased expressions of HDAC6 and KLF5, and increased MAFF expression (*p < 0.05). The sh-HDAC6 + oe-KLF5 group had enhanced KLF5 expression, but the expressions of HDAC6 and MAFF showed no significant difference with those in sh-HDAC6 + oe-NC group (Figures 4(A,B), ^#^*p* < 0.05). The measurement on fibrosis and inflammation related proteins showed sh-HDAC6 can attenuate fibrosis and inflammatory response in renal epithelial cells, while such effect can be abolished by oe-KLF5 (Figures 4(C,D), ^#^*p* < 0.05). Taken together, HDAC6 exacerbated fibrosis and inflammation in renal epithelial cells by regulating the MAFF/KLF5 axis.

### 3.5. HDAC6 mediates fibrosis and inflammation in LN mice by regulating the MAFF/KLF5 axis

**Figure 5.**
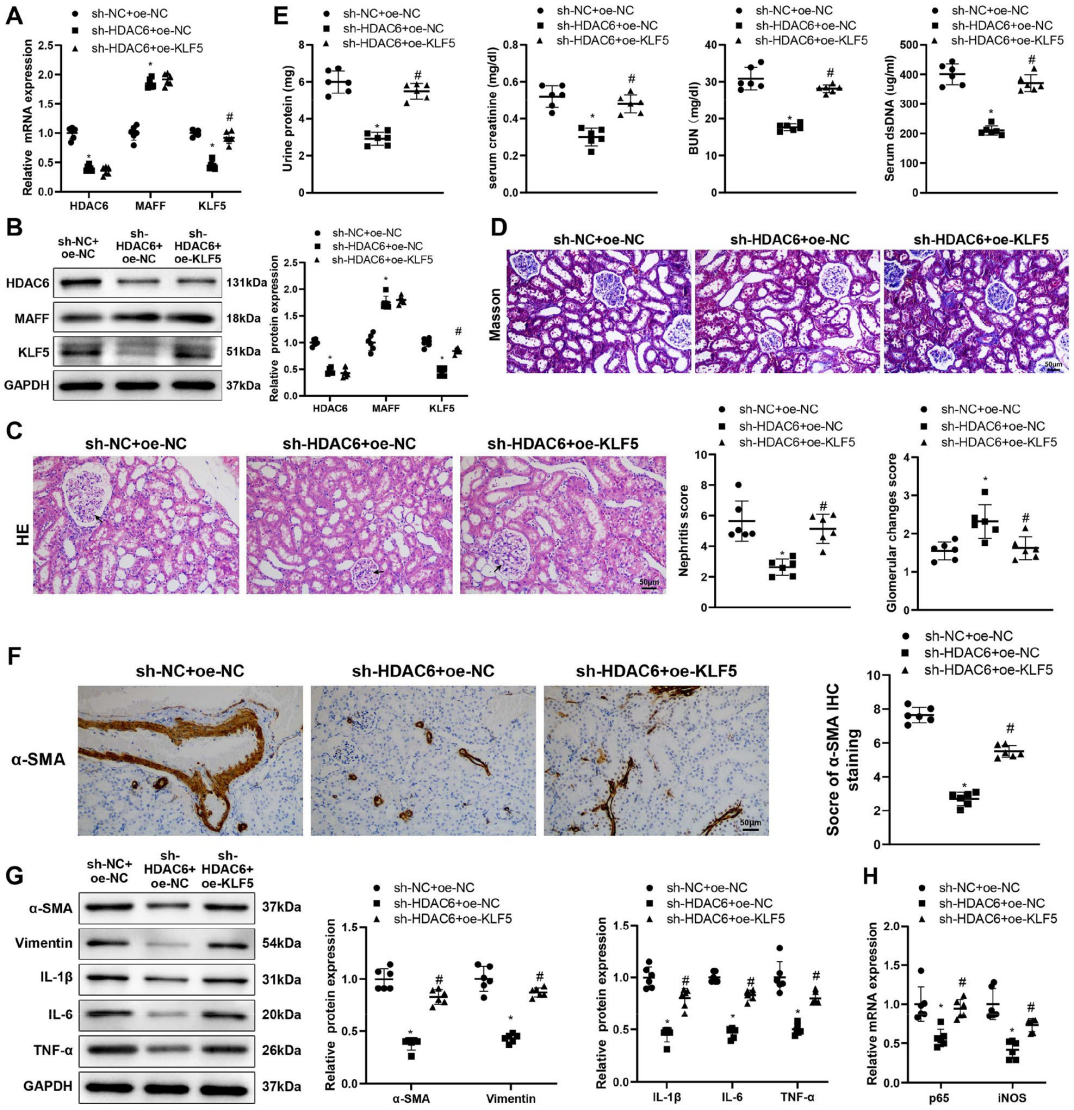
HDAC6 mediates fibrosis and inflammation in LN mice *via* regulating MAFF/KLF5 axis, (A,B) expressions of HDAC6, MAFF, and KLF5 were detected by RT-qPCR and Western blot; (C,D) morphological observation after H&E and Masson staining; (E) biochemical analysis detecting proteinuria, SCr, BUN, and dsDNA levels; (F) α-SMA expressions were detected by immunofluorescence; (G) Western blot was applied to detect the expressions of fibrosis and inflammation related proteins, α-SMA, Vimentin, IL-1β, IL-6, and TNF-α; (H) the mRNA levels of p65 and iNOS were measured using RT-qPCR. Data were expressed as mean ± standard deviation. *N* = 6 for animal experiments. One-way analysis of variance and Tukey’s multiple comparisons test were used for comparison among multiple groups. *p < 0.05, when compared with sh-NC + oe-NC group; ^#^*p* < 0.05, when compared with sh-HDAC6 + oe-NC group. BUN: blood urea nitrogen; dsDNA: double-stranded DNA; SCr: serum creatinine; LN: lupus nephritis.

*In vivo* experiments were conducted in MRL/lpr mice, in which sh-HDAC6 or/and oe-KLF5 were injected. Western blot demonstrated that the expressions of HDAC6 and KLF5 were suppressed and MAFF expression was increased in sh-HDAC6 + oe-NC group compared with those in sh-NC + oe-NC group (*p < 0.05). The expression of KLF5 was enhanced in sh-HDAC6 + oe-KLF5 group, but the expressions of HDAC6 and MAFF showed no significant difference with that in sh-HDAC6 + oe-NC group (Figures 5(A,B), *p < 0.05). H&E and Masson staining on renal tissues showed the renal fibrosis and inflammation in MRL/lpr mice can be alleviated by HDAC6 suppression, and enhanced by further KLF5 overexpression (Figures 5(C,D), ^#^*p* < 0.05). Consistent results were obtained by biochemical analysis on proteinuria, SCr, BUN, and dsDNA levels, immunofluorescence on α-SMA, western blot on fibrosis and inflammatory related proteins, and RT-qPCR on mRNA levels of p65 and iNOS (Figures 5(E–H), ^#^*p* < 0.05). Overall, HDAC6 aggravated renal fibrosis and inflammation in mice with LN *via* the MAFF/KLF5 signaling axis.

## 4. Discussion

Renal fibrosis is an event in LN of primary importance in the early stage to identify the risks of developing ESRD and associated with accumulated ECM component in response to chronic injury in the kidney [38]. Additionally, oxidative stress and inflammation play a central role in renal fibrosis, forming a vicious cycle: oxidative stress induces inflammation through a variety of molecular mechanisms, whereas inflammation triggers oxidative stress by activating white blood cells and resident cells to produce reactive oxygen and nitrogen species [39]. Pediatric LN is one of the refractory secondary kidney diseases in childhood, presented with various manifestations, which makes it much difficult to be diagnosed at the first time [40]. This study explored the possible effect and involvement of HDAC6 in regulating renal fibrosis and inflammation in LN using both *in vivo* and *in vitro* experiments and highlighted the promotive effect of HDAC6 on LN progression through regulating the MAFF/KLF5 axis.

The renal fibrosis is associated with the activation of renal interstitial fibroblasts and the accumulation of excessive ECM [20], caused by injuries of renal tubular epithelial cells [4]. As a chronic inflammatory process, renal fibrosis is a well-recognized determinant factor in defining response to therapy and renal prognosis in LN [41], along with the secretion of inflammatory cytokines [42]. The elevation of HDAC6 expression would lead to the degradation of protein aggregates, which was found in various neurodegenerative disorders, including Alzheimer’s and Parkinson’s diseases [43]. The regulation of transforming growth factor β1 (TGF-β1) by HDAC6 is of great importance for the pathogenesis of fibrotic diseases through epithelial-mesenchymal transitions (EMT) [44]. The same study also identified the suppression of HDAC6 inhibitors against lung fibrosis in mouse models. In addition to that, HDAC6 inhibition attenuated renal tubular injury in chronic kidney disease, evidenced by attenuated proteinuria progression, and diminished tubulointerstitial collagenous matrix deposition [45]. Consistent with this finding, this study demonstrated the increased HDAC6 expression in LN mouse and cell models, and the induction of HDAC6 contributed to the accumulation of vimentin and α-SMA, as showed by IHC and IF. A similar observation was found in a study on obstructive nephropathy, which concluded that inhibition of HDAC6 significantly reduced the expression levels of α-SMA, a hallmark of myofibroblasts *in vivo* and *in vitro* [20]. In addition to that, the increase of vimentin contributes to the secretion of ECM, which can be abolished by HDAC6 inhibition [46]. All evidences supported the regulation of HDAC6 on renal fibrosis in LN. In renal ischemia-reperfusion injury, HDAC6 was reported to regulate renal protective protein Klotho through deacetylation of H3K9 [47]. Therefore, we speculate the regulation of HDAC6 on LN may achieve through deacetylation.

MAFF is a transcription factor that has close association with inflammation and stress [48], and belongs to the small MAF proteins of the MAF family, which only has a basic region leucine zipper (bZip) domain [49]. MAFF was found to serve as a hypoxia gene to promote tumor invasion and metastasis [50]. Analysis on datasets of GSE51921 and GSE112493 identified the deregulated expression of MAFF in LN, suggesting the possible implication of MAFF in LN. Our *in vitro* experiments on renal epithelial cells showed MAFF overexpression can attenuate the inflammation and fibrosis response, evidenced by suppressed pro-inflammatory cytokines (IL-1β, IL-6, and TNF-α) and fibrosis related protein (α-SMA and Vimentin) expressions in IL-33 treated cells. RNAseq analysis in a previous study also reported MAFF as one of the downregulated genes in chronic kidney disease mouse models [51]. As a transcription factor associated with inflammation, MAFF can be induced by inflammatory cytokines and oxidative stress [36]. Meanwhile, MAFF can also work as a pro-inflammatory factor in stimulation of LPS [52]. All the above suggest the casual role of MAFF in inflammatory process. UCSC database found the possible interaction of MAFF and HDAC6, which was further supported by ChIP assay. Rescue assays also confirmed the regulation of HDAC6 on MAFF *via* deacetylation to further mediate fibrosis and inflammation process in renal epithelial cells.

One important question is whether MAFF can regulate its downstream target gene to mediate LN progression. JASPER database found the binding sites between MAFF and KLF5. In our previous study, KLF5 was found to act as a suppressor in renal fibrosis through regulating MX1 [23]. Consistent with our previous results, the KLF5 expression in this study was found to be increasingly presented in renal tissues of MRL/lpr mouse and in renal epithelial cells, whose regulation on renal fibrosis and inflammation in LN was further confirmed by *in vivo* experiments. In agreement with this observation, the implication of KLF5 on renal related diseases, including CKD, diabetic nephropathy can be found in several studies [53,54]. Nevertheless, the KLF5 haploinsufficiency in bilateral ureteral obstruction mouse was found to ameliorate renal injury and dysfunction, despite its enhancement of renal fibrosis [55]. The inconsistence of anti-fibrotic or pro-fibrotic role of KLF5 may be explained by different stimulants/cell types/disease models and may involve different organs, which further emphasizes the needs of more validation by future studies. This study also identified the negative regulation of MAFF on KLF5 transcription in LN mouse and cell models through rescue experiments, which found the suppressive effect of HDAC6 inhibition on renal fibrosis and inflammation response can be abolished by KLF5 overexpression, suggested the involvement of the HDAC6/MAFF/KLF5 axis in LN.

## 5. Conclusion

In conclusion, our results concluded HDAC6 can regulate MAFF expression through deacetylation and subsequently inhibit the suppressive effect of MAFF on KLF5 expression to increase KLF5 expression. The HDAC6/MAFF/KLF5 axis in mediating fibrosis and inflammation in IL-33 induced renal epithelia cells was further confirmed by *in vivo* experiments, suggesting the implication of this axis in LN progression. As we mentioned before, more evidence are in need to validate the result of this study due to the ­complication of fibrosis and inflammation process. Al­­though treatments of LN are mandatory to minimize its impact on SLE patients, any theoretical basis should be validated with caution before any clinical application can be proposed.

## Supplementary Material

Supplemental Material
