# Supplementary material for: HDAC6 promotes inflammation in lupus nephritis mice by regulating transcription factors MAFF and KLF5 in renal fibrosis
Source: Ren Fail. 2024 Oct 16;46(2):2415517. doi: 10.1080/0886022X.2024.2415517 (PMC11485742; doi:10.1080/0886022X.2024.2415517)
Supplement: Supplemental Material [file IRNF_A_2415517_SM2994.docx]

**
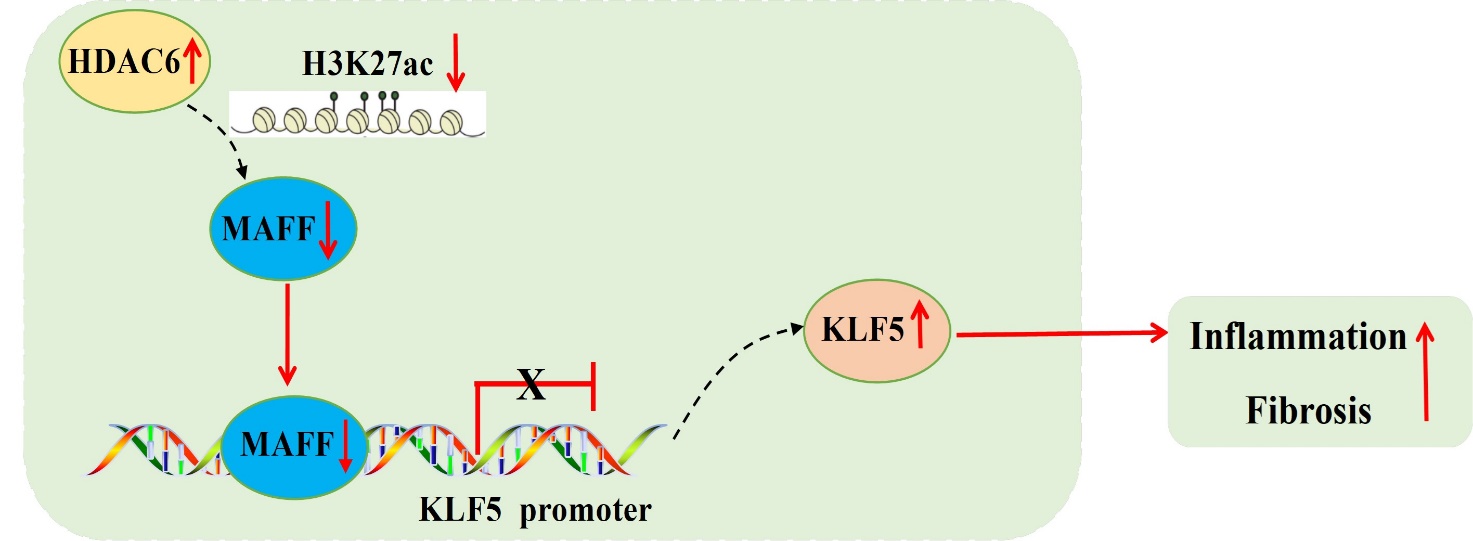
**

**Supplementary Figure 1** Mechanisms by which increased HDAC6 activity promotes renal fibrosis.


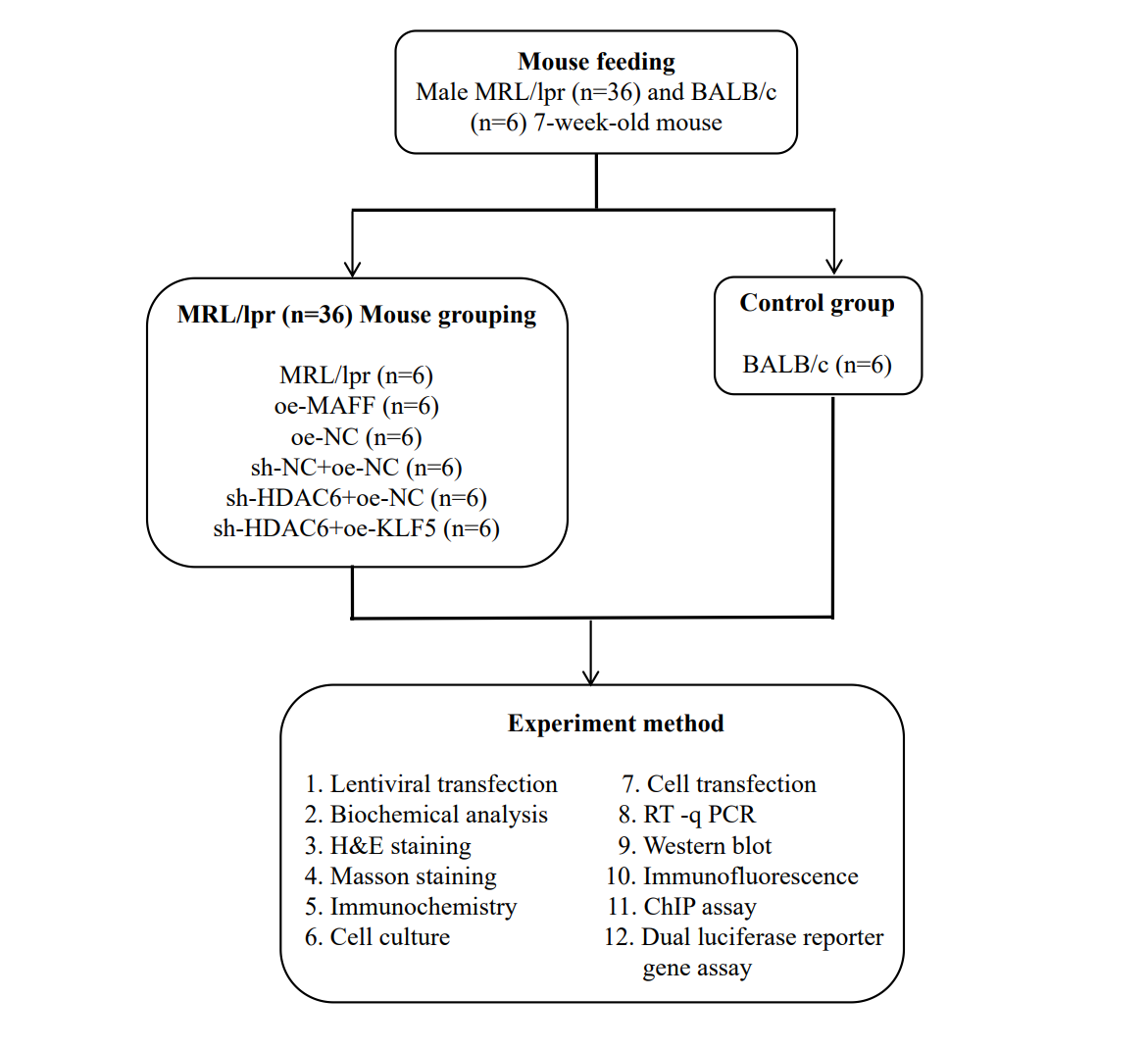


**Supplementary Figure 2** Study flow chart.

**
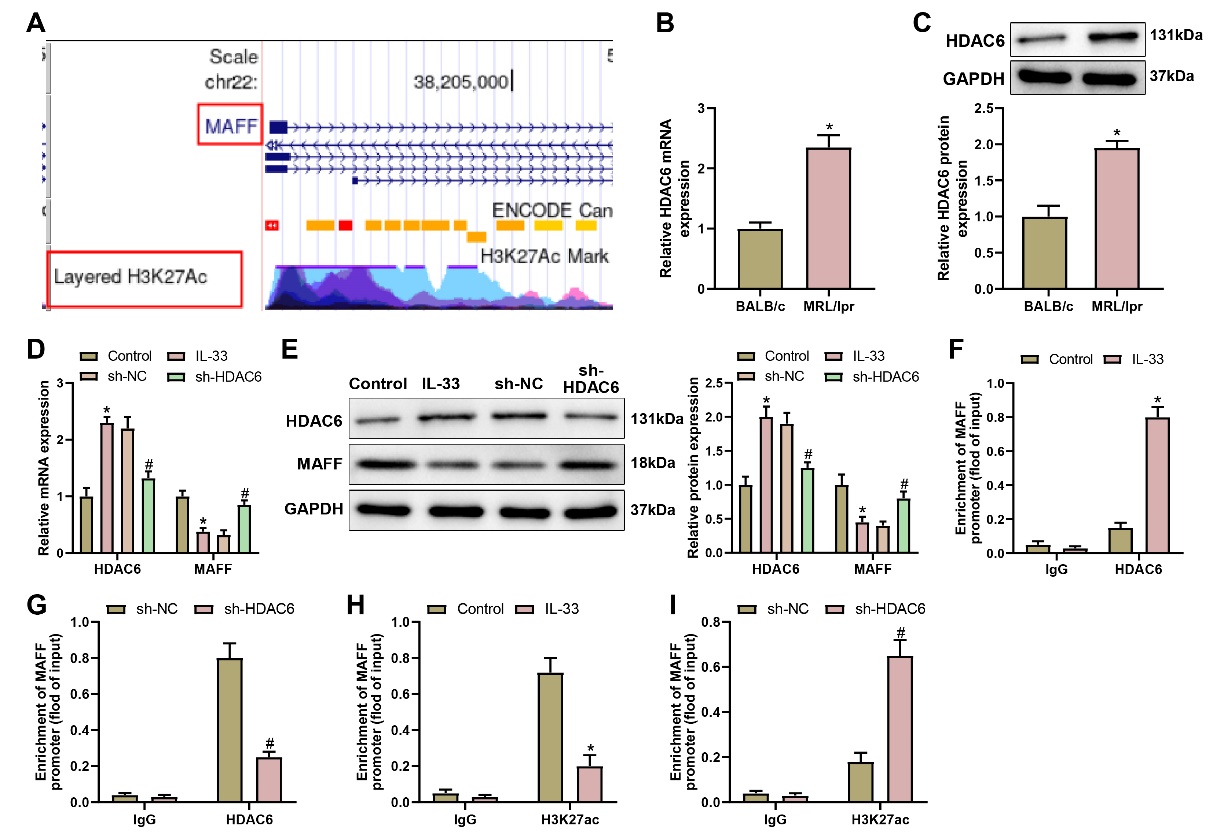
**

**Supplementary figure 3** HDAC6 decreases MAFF expression through deacetylation

A, H3K27ac levels in the promoter of MAFF in UCSC database; B-C, RT-qPCR and western blot were applied to detect the expressions of MAFF in renal tissues; D-E, After sh-HDAC6 was transfected into renal epithelial cells, the expressions of MAFF were detected by RT-qPCR and western blot; F-G, ChIP assay detected the enrichment of HDAC6 in the MAFF promoter after corresponding treatment; H-I, ChIP assay detected the enrichment of H3K27ac in the MAFF promoter after corresponding treatment. Data were expressed as mean ± standard deviation. N = 6 for mice experiments (B-C) and N = 3 for cellular experiments (D-I). *T* test was used for analysis between two groups. One-way analysis of variance and Tukey's multiple comparisons test were used for comparison among multiple groups. **p* < 0.05, when compared with control or BALB/c group; ^#^*p* < 0.05, when compared with sh-NC group.

**
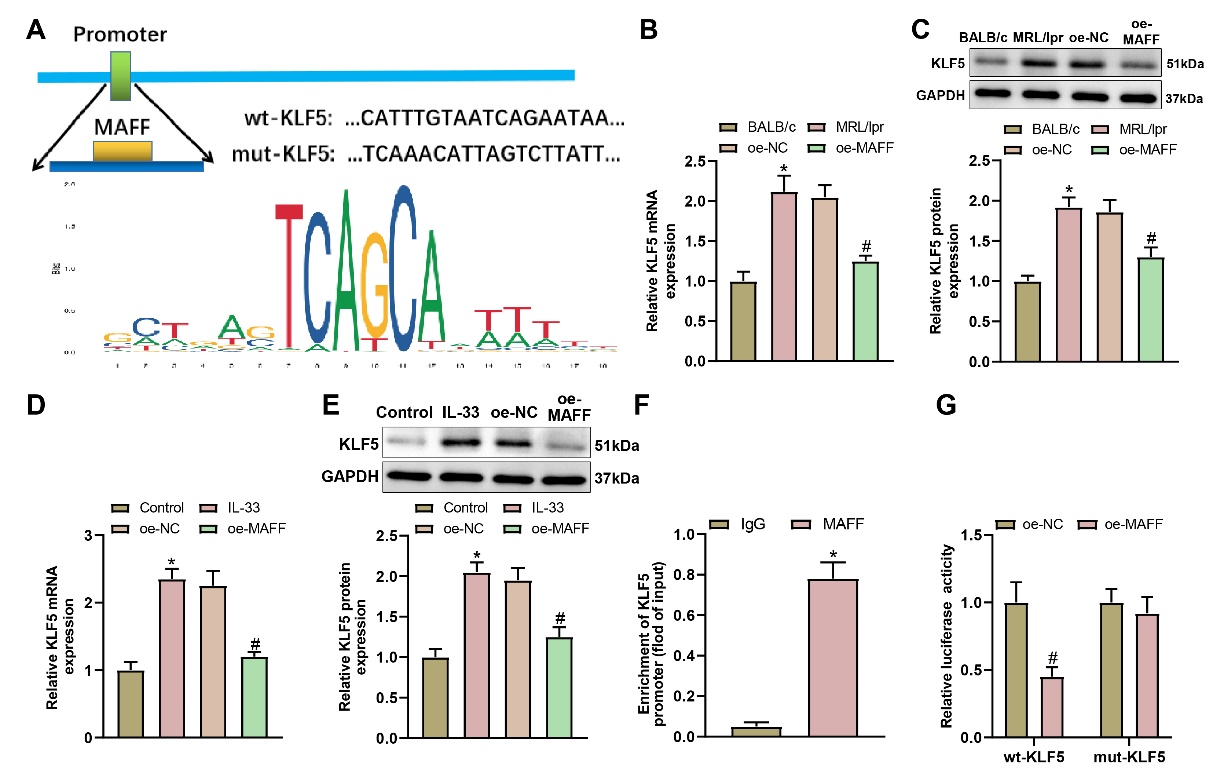
**

**Supplementary figure 4** MAFF regulates KLF5

A, the binding sites of MAFF with KLF5 predicted by Jaspar; B-C, KLF5 expression in renal tissues was detected by RT-qPCR and western blot; D-E, KLF5 expression in renal epithelial cells was detected by RT-qPCR and western blot; F-G, ChIP assay and dual luciferase reporter gene assay verified the binding of MAFF with KLF5. Data were expressed as mean ± standard deviation. N = 6 for mice experiments (B-C) and N = 3 for cellular experiments (D-G). *T* test was applied for comparison between two groups. One-way analysis of variance and Tukey's multiple comparisons test were used for comparison among multiple groups. **p* < 0.05, when compared with BALB/c, control, or IgG group; ^#^*p* < 0.05, when compared with oe-NC group.

**Table 1. Primer sequences**

| Name of primer | Sequences (5'-3') |
| --- | --- |
| HDAC6-F | TGCAGGAGGTGGAGTTGAGT |
| HDAC6-R | GAAGAATCTTGGCCGGTGGA |
| MAFF-F | GTTCTCCTAGGCTGAGGATGTG |
| MAFF-R | ATCAGCGCTTCATCCGACA |
| KLF5-F | CACCGGATCTAGACATGCCC |
| KLF5-R | ACGTCTGTGGAACAGCAGAG |
| GAPDH-F | CCCTTAAGAGGGATGCTGCC |
| GAPDH-R | ACTGTGCCGTTGAATTTGCC |
| p65-F | CCTCGGGACAAACAGCCTC |
| p65-R | TGCTTCGGCTGTTCGATGAT |
| iNOS-F | CAACAGGGAGAAAGCGCAAA |
| iNOS-R | GGCCTTGTGGTGAAGAGTGT |

F, forward; R, reverse.
